# Associations between childhood family emotional health, fronto-limbic grey matter volume, and saliva 5mC in young adulthood

**DOI:** 10.1186/s13148-021-01056-y

**Published:** 2021-03-31

**Authors:** J. R. Pfeiffer, Angela C. Bustamante, Grace S. Kim, Don Armstrong, Annchen R. Knodt, Karestan C. Koenen, Ahmad R. Hariri, Monica Uddin

**Affiliations:** 1 Department of Psychology, University of Illinois at Urbana-Champaign, Urbana, IL USA; 2 Carl R. Woese Institute for Genomic Biology, Urbana, IL USA; 3 Division of Pulmonary and Critical Care Medicine, Department of Internal Medicine, University of Michigan Medical School, Ann Arbor, MI USA; 4 Medical Scholars Program, University of Illinois College of Medicine, Urbana, IL USA; 5 Department of Psychology and Neuroscience, Duke University, Durham, NC USA; 6 Department of Epidemiology, Harvard School of Public Health, Boston, MA USA; 7 Laboratory of NeuroGenetics, Duke University, Durham, NC USA; 8 Genomics Program, College of Public Health, University of South Florida, 3720 Spectrum Blvd., Suite 304, Tampa, FL USA

**Keywords:** Peripheral epigenetics, Neuroimaging, Family emotional health, Adverse social environment, Biological embedding, Fronto-limbic

## Abstract

**Background:**

Poor family emotional health (FEH) during childhood is prevalent and impactful, and likely confers similar neurodevelopmental risks as other adverse social environments. Pointed FEH study efforts are underdeveloped, and the mechanisms by which poor FEH are biologically embedded are unclear. The current exploratory study examined whether variability in 5-methyl-cytosine (5mC) and fronto-limbic grey matter volume may represent pathways through which FEH may become biologically embedded.

**Results:**

In 98 university students aged 18–22 years, retrospective self-reported childhood FEH was associated with right hemisphere hippocampus (*b* = 10.4, *p* = 0.005), left hemisphere amygdala (*b* = 5.3, *p* = 0.009), and right hemisphere amygdala (*b* = 5.8, *p* = 0.016) volumes. After pre-processing and filtering to 5mC probes correlated between saliva and brain, analyses showed that childhood FEH was associated with 49 5mC principal components (module eigengenes; MEs) (*p*_range_ = 3 × 10^–6^ to 0.047). Saliva-derived 5mC MEs partially mediated the association between FEH and right hippocampal volume (Burlywood ME indirect effect *b* = − 111, *p* = 0.014), and fully mediated the FEH and right amygdala volume relationship (Pink4 ME indirect effect *b* = − 48, *p* = 0.026). Modules were enriched with probes falling in genes with immune, central nervous system (CNS), cellular development/differentiation, and metabolic functions.

**Conclusions:**

Findings extend work highlighting neurodevelopmental variability associated with adverse social environment exposure during childhood by specifically implicating poor FEH, while informing a mechanism of biological embedding. FEH-associated epigenetic signatures could function as proxies of altered fronto-limbic grey matter volume associated with poor childhood FEH and inform further investigation into primarily affected tissues such as endocrine, immune, and CNS cell types.

**Supplementary Information:**

The online version contains supplementary material available at 10.1186/s13148-021-01056-y.

## Background

Children can be exposed to an array of adverse social environments (ASEs) throughout their development, such as stressful life events (SLEs) and trauma. In addition, growing up in a low socioeconomic status (SES) household, or a household in which a caregiver is diagnosed with psychopathology, conveys risk towards potentially adverse exposures. Caregiver psychopathology is of particular interest to the current research due to its wide-reaching effects throughout the family unit, and its prevalence in the United States; it is estimated that ~ 12.8 million parents suffer yearly from some form of mental illness (18.2%), and that ~ 2.7 million parents suffer yearly from a *serious* mental illness (3.8%) [1]. The psychological effects of living with caregivers with a mental illness can be notably deleterious. Children of caregivers with major depressive disorder (MDD), for example, are subject to elevated risk of more hostile, negative, and withdrawn parenting [2]. Estimates range from two to 13 times increased risk for children to develop either their caregiver’s mental illness or a mental illness different from their caregiver’s [3]. Children growing up in these conditions are also more likely to develop internalizing or externalizing behavioral problems, as well as social, cognitive, and academic difficulties [4, 5]. An extension of caregiver psychopathology exposure is family emotional health (FEH). Importantly, the mechanisms by which poor FEH are biologically embedded and produce these adverse outcomes are unclear.

The neuroimmune network hypothesis is one framework used to explain the physiological mechanisms via which ASEs and caregiver mental illness affect the mental health of offspring. The neuroimmune network hypothesis focuses on the integrated, bi-directional network of the central nervous system (CNS) and the immune system [6]. It posits that exposure to ASEs during childhood, an especially plastic window of development [7], impacts communication between peripheral inflammatory signals and fronto-limbic brain regions (i.e. prefrontal cortex, hippocampus, and amygdala). These brain regions support threat, reward, executive control, memory, and adaptive behavioral/emotional responses, among others [8]. Importantly, these functions are impaired in numerous affective and stress-related mental illnesses [9–12]. These inflammatory signals disrupt the inter-dependent functions of the fronto-limbic pathways, leading to altered behavioral states, and the pre-disposition to develop aberrant stress responses later in life [13]. These concepts are supported by a significant body of research that has shown immune system [14–16], hypothalamic–pituitary–adrenal (HPA)-axis [17, 18], and fronto-limbic pathway [18–22] associations with ASE exposure. More specifically, researchers have shown that childhood exposure to factors similar to poor FEH, such as maternal support and supportive/hostile parenting, are associated with lower hippocampus and amygdala grey matter volume later in life [23, 24]. Observed in association with ASE exposures, the signatures of morphometric variability within the fronto-limbic pathway are regarded as neural correlates of these exposures [18–24], and as neural endophenotypes of psychiatric illness [25–27].

The molecular mechanisms by which ASEs, including caregiver mental illness, become biologically embedded in the CNS are currently under investigation [28], and research has pointed to the importance of epigenetics, particularly 5-methyl-cytosine (5mC) levels, in this process [29, 30]. 5mC serves as a mediator of gene by environment interaction [31–34], but it remains challenging to measure epigenetics in the living human brain—the primary etiologic tissue of interest in regard to mental health-related outcomes. This limitation has prompted investigation into epigenetic measures collected from peripheral tissue, such as saliva, which may serve as proxies for etiological tissue. Previous studies have provided a framework for the use of peripheral tissues in epigenome-wide association studies (EWAS) and support the potential use of peripheral 5mC as a proxy for etiological tissue 5mC [35]. Further bolstering the notion that peripheral 5mC is an efficacious proxy for etiological tissue 5mC, is research showing that peripheral epigenetic measures can index changes in the HPA-axis [36, 37], immune system [38, 39], and the CNS [40–42]. However, these relationships do not directly indicate association between peripheral epigenetic measures and CNS-relevant endophenotypes of psychopathology. On this note, studies have used human structural and functional neuroimaging data in tandem with epigenetic measures but have primarily utilized candidate gene approaches. Measuring peripheral 5mC of the *SLC6A4* [43–45], *NR3C1* [46, 47], *FKBP5* [48], and *SKA2* [49, 50] genes, these studies have investigated associations between peripheral 5mC and variability in the structure and function of the frontal cortex, hippocampus, and amygdala. Findings suggest that locus-specific peripheral 5mC can index CNS structural alterations [43–50], and may statistically mediate ASE-induced CNS structural alterations [50].

Despite the evidence that peripheral 5mC can index CNS-related phenotypes, to date few studies, to our knowledge, have examined these relations in a hemisphere-specific manner within the brain. Importantly, numerous aspects of human behavior and biology are subject to hemisphere-specific brain lateralization [51–53]. This, coupled with evidence of hemisphere-specific fronto-limbic variability in association with ASEs in humans [18–24], provides a solid framework to address the potential associations of poor FEH with *hemisphere-specific* volume measurements. Beyond the aforementioned reports, studies of poor FEH or caregiver mental illness on CNS structure are sparse and limited to biological offspring of parents with genetically heritable psychopathology, although some investigate associations of exposure with outcome on a hemisphere-specific basis [54, 55]. These types of ASEs are also associated with changes in cell type-specific and tissue-specific 5mC [56], further highlighting the potential biologic embedding of these adverse exposures.

However, to our knowledge, investigations into the role of poor FEH in association with neural endophenotypes of psychopathology development have yet to be reported, and therefore, the magnitude of risk associated with poor childhood FEH has not been elucidated. In addition, investigations into the potential epigenetic mechanisms explaining the biological embedding of poor FEH have yet to be carried out.

To address these gaps in the field, the current exploratory study applied genome-scale approaches to assess whether saliva-derived 5mC measurements might index CNS endophenotypes of psychopathology in a sample of 98 young adult volunteers. We were specifically interested whether saliva-derived 5mC principal components (module eigengenes; MEs) might statistically mediate the relationship between poor FEH and hemisphere-specific fronto-limbic grey matter volume, while controlling for age, sex, cellular heterogeneity, genomic ancestry, past year SLEs, and total brain volume (TBV). Such a result may serve as a peripheral proxy of such CNS variability, while informing a *potential* biological mechanism of physiological embedding. Based on previous work, we hypothesized that identified 5mC modules would be enriched with 5mC probes falling in genes with HPA-axis, immune system, and CNS-relevant gene ontology (GO) functions.

## Results

### Study participants

Descriptive statistics for demographic, psychosocial, and neuroimaging variables in study participants are shown in Table 1. FEH ranged from 34 to 70; the mean in the study sample was 60 (± 8.5). To compare differences between hemisphere volumes of mPFC, dlPFC, amygdala, and hippocampus we performed paired-sample *t*-tests of each brain region. We found significant differences between left and right hemisphere volume in dlPFC (mean_Left_ = 11,980 mm^3^, mean_Right_ = 10,639 mm^3^, *t* = 9.1, *p* = 1 × 10^−14^), amygdala (mean_Left_ = 1655 mm^3^, mean_Right_ = 1859 mm^3^, *t* = − 12.5, *p* = 0.005), and hippocampus (mean_Left_ = 4650 mm^3^, mean_Right_ = 4741 mm^3^, *t* = − 2.9, *p* = 2 × 10–16), but no difference in mPFC (*p* > 0.05).

**Table 1:** Demographic, psychosocial, and neuroimaging summary stats for the current sample (*n* = 98)

Characteristic	Description	Value
Age	Mean [SD] (range)	19.8 [1.2] (18–22)
Sex	Male	31%
	Female	69%
Self-reported race/ethnicity	Caucasian/White	49%
	African American/Black	6%
	Asian	22%
	American Indian	13%
	Bi- or multiracial	0%
	Other	9%
Cumulative perceived impact of past year SLEs	Mean [SD] (range)	11 [7.6] (1–42)
Family emotional health (FEH)	Mean [SD] (range)	60 [8.5] (34–70)
Left hippocampus volume (mm^3^)	Mean [SD] (range)	4650 [446] (3267–5679)
Right hippocampus volume (mm^3^)	Mean [SD] (range)	4741 [392] (3825–6031)
Left dlPFC volume (mm^3^)	Mean [SD] (range)	11,980 [1785] (7308–16,007)
Right dlPFC volume (mm^3^)	Mean [SD] (range)	10,639 [1585] (7426–14,819)
Left mPFC volume (mm^3^)	Mean [SD] (range)	5745 [853] (3871–8686)
Right mPFC volume (mm^3^)	Mean [SD] (range)	5790 [717] (4238–7442)
Left amygdala volume (mm^3^)	Mean [SD] (range)	1655 [186] (1292–2157)
Right amygdala volume (mm^3^)	Mean [SD] (range)	1859 [210] (1371–2417)
Total brain volume (mm^3^)	Mean [SD] (range)	1.2 × 10^6^ [1.2 × 10^5^] (9.0 × 10^5^–1.5 × 10^6)^

### Correlation analyses

**Fig. 1:**
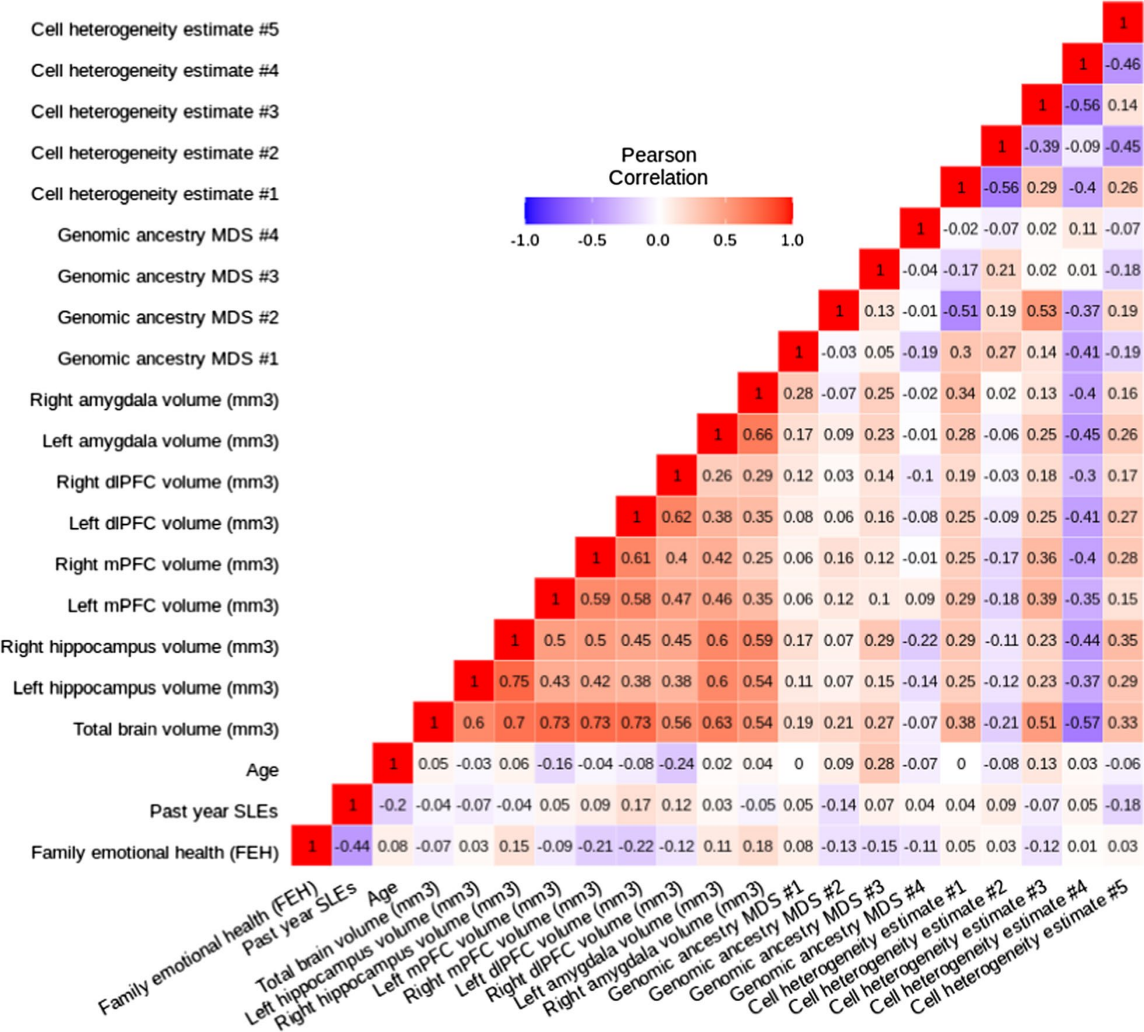
Pearson correlation heat map of variables used throughout the current analyses. **a** A strong negative relationship was observed between FEH and past year SLEs (Pearson’s correlation: *r* = − 0.44, *p* = 7 × 10^–6^). Strong positive relationships are also observed between hemisphere-specific brain regions (Pearson’s correlation *r* range: 0.26–0.75)

Pearson correlations between variables used in the current study were mapped (Fig. 1). Of note, a moderate negative association was observed between FEH and past year SLEs (Pearson’s correlation: *r* = − 0.44, *p* = 7 × 10^–6^).

### FEH predicts hemisphere-specific brain region volume (BRV)

FEH was positively associated with right hippocampus (*b* = 10.4, SE = 3.6, *t* = 2.9, *p* = 0.005), left amygdala (*b* = 5.3, SE = 2.0, *t* = 2.7, *p* = 0.009), and right amygdala volumes (*b* = 5.8, SE = 2.3, *t* = 2.4, *p* = 0.016). These significant relationships were also observed in models without controlling for the covarying effect of TBV (right hippocampus *p* = 0.015; left amygdala *p* = 0.018; right amygdala *p* = 0.023). FEH was not associated with left hippocampus (*p* = 0.62), left dlPFC (dorsolateral PFC) (*p* = 0.10), right dlPFC (*p* = 0.62), left mPFC (medial PFC) (*p* = 0.98), or right mPFC volume (*p* = 0.09). In controlling for seventy-two tests at false discovery rate (FDR) = 0.10, all three brain regions with *p* < 0.05 were Benjamini Hochberg (BH)-significant (Table 2). Regions associated with FEH were carried into following analyses.

**Table 2:** Family emotional health (FEH) predicts hemisphere-specific brain region volume (BRV) (*n* = 98)

L. hipp vol. (mm^3^)	Adj. *R*^2^	RSE	*b*	SE	R. hipp vol. (mm^3^)	Adj. *R*^2^	RSE	*b*	SE
Model	0.325***	369.2			Model	0.532***	255.9		
FEH			2.6	5.2	FEH			10.4**	**3.6**
Sex (female)			− 60.6	114.9	Sex (female)			− 88.5	79.7
Age			− 32.1	33.2	Age			− 8.4	23.0
Cumulative impact of past year SLE			− 2.5	5.9	Cumulative impact of past year SLE			4.1	4.1
Total brain volume (mm^3^)			0.002***	**0.0005**	Total brain volume (mm^3^)			0.002***	**0.0003**

L. dlPFC vol. (mm^3^)	Adj. *R*^2^	RSE	*b*	SE	R. dlPFC vol. (mm^3^)	Adj. *R*^2^	RSE	*b*	SE
Model	0.564***	1182.0			Model	0.344**	1254.0		
FEH			− 27.9	16.7	FEH			− 8.8	17.7
Sex (female)			366.8	367.9	Sex (female)			219.2	390.3
Age			− 82.5	106.3	Age			− 323.2**	**112.8**
Cumulative impact of past year SLE			28.3	18.9	Cumulative impact of past year SLE			10.9	20.0
Total brain volume (mm^3^)			0.013***	**0.0015**	Total brain volume (mm^3^)			0.008***	**0.0016**

L. mPFC vol. (mm^3^)	Adj. *R*^2^	RSE	*b*	SE	R. mPFC vol. (mm^3^)	Adj. *R*^2^	RSE	*b*	SE
Model	0.559***	557.6			Model	0.539***	479.1		
FEH			0.2	7.9	FEH			− 11.6	6.8
Sex (female)			159.9	173.6	Sex (female)			53.1	149.1
Age			− 102.9*	50.2	Age			− 11.6	43.1
Cumulative impact of past year SLE			4.8	8.9	Cumulative impact of past year SLE			5.7	7.7
Total brain volume (mm^3^)			0.006***	**0.0007**	Total brain volume (mm^3^)			0.005***	**0.0006**

L. amygdala vol. (mm^3^)	Adj. *R*^2^	RSE	*b*	SE	R. amygdala vol. (mm^3^)	Adj. *R*^2^	RSE	*b*	SE
Model	0.404***	139.2			Model	0.356***	166.2		
FEH			5.2**	**2.0**	FEH			5.8*	**2.3**
Sex (female)			− 34.5	43.3	Sex (female)			− 34.3	51.7
Age			− 3.6	12.5	Age			− 5.1	15.0
Cumulative impact of past year SLE			3.5	2.2	Cumulative impact of past year SLE			0.7	2.7
Total brain volume (mm^3^)			0.001***	**0.0002**	Total brain volume (mm^3^)			0.001***	**0.0002**

P: BH-significant (bolded)

Four genomic ancestry multi-dimensional scaling measures were included in each model, but are not shown here

*RSE* relative standard error, *adj* adjusted, *b* estimate, *SE* standard error, *L* left, *R* right, *hipp* hippocampus, *vol* volume

***P < 0.001; **P < 0.01; *P < 0.05

### FEH predicts ME values

FEH was associated with 49 MEs (*b*_min_ = − 0.006, *b*_max_ = 0.006, *p*_min_ = 3 × 10^–6^, *p*_max_ = 0.047). Twenty-nine out of 49 MEs achieved BH-significance, including the Burlywood and Pink4 MEs, taking 194 tests into account at FDR = 0.10 (Additional file 1: Table S1).

### ME values predict hemisphere-specific BRV

**Fig. 2:**
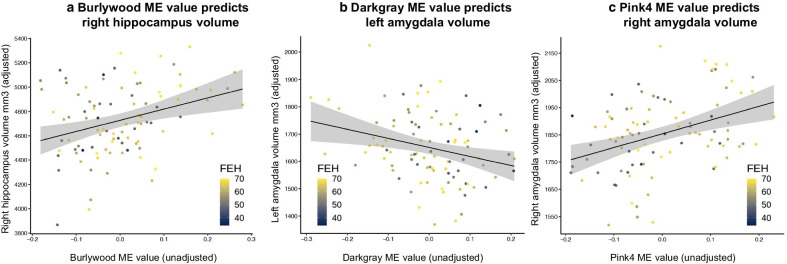
ME values are associated with high right hippocampal, low left amygdala, and high right amygdala volume. BRVs values shown are adjusted by covariates. Covariates across all models: age, sex, four genomic ancestry measures, past year SLEs, and total brain volume. The line of best fit (via least squares) is shown with a grey 95% SE confidence range. **a** High Burlywood ME value is associated with high right hippocampal volume (*b* = 874.2, SE = 252.1, *t* = 3.5, *p* = 0.0008). **b** High Darkgray ME value is associated with low left amygdala volume (*b* = − 374.2, SE = 140.1, *t* = − 2.7, *p* = 0.009). **c** High Pink4 ME value is associated with high right amygdala volume (*b* = 467.5, SE = 165.8, *t* = 2.8, *p* = 0.006)

Forty-nine MEs associated with FEH were tested for association with right hippocampus, left amygdala, and right amygdala volumes (Additional file 1: Table S2). Seven MEs were associated with right hippocampus volume, four of which were BH-significant: Burlywood (*b* = 874.2, SE = 252.1, *t* = 3.5, *p* = 8 × 10^–4^) (Fig. 2a), Darkolivegreen1 (*b* = 770.0, SE = 258.6, *t* = 3.0, *p* = 0.004), Thistle2 (*b* = 728.1, SE = 261.8, *t* = 2.8, *p* = 0.007), and Chocolate2 (*b* = − 713.3, SE = 259.3, *t* = − 2.8, *p* = 0.007). The Darkgray ME (*b* = − 374.2, SE = 140.1, *t* = − 2.7, *p* = 0.009) (Fig. 2b) was negatively associated with left amygdala volume, in addition to the Darkolivegreen ME (*b* = − 300.6, SE = 142.2, *t* = − 2.1, *p* = 0.037). The Lavenderblush2 ME was positively associated with left amygdala volume (*b* = 295.1, SE = 144.5, *t* = 2.0, *p* = 0.044). Finally, the Pink4 ME (*b* = 467.5, SE = 165.8, *t* = 2.8, *p* = 0.006) (Fig. 2c) was positively associated with right amygdala volume. In controlling for 49 tests within each of the three BRVs at FDR = 0.10, only the aforementioned MEs associated with right hippocampus volume were BH-significant.

### ME mediation

Eleven MEs were tested for mediation between FEH and brain region volumes (BRVs)**.** The Burlywood ME was a partial *statistical* mediator between FEH and right hippocampus volume (*b*_TE_ = − 366, *p* = 8 × 10^–4^; *b*_IDE_ = − 111, *p* = 0.014; *b*_DE_ = − 254, *p* = 0.037). The total effect (TE) indicated that right hippocampal volume was 366 mm^3^ less under poor FEH conditions compared to high FEH conditions, while the indirect effect (IDE) of the Burlywood ME was accountable for 111 mm^3^ (30%) of that effect. Without controlling for the covarying effect of TBV, the Burlywood ME was a full mediator (*b*_TE_ = − 376, *p* = 0.006; *b*_IDE_ = − 114, *p* = 0.031; *b*_DE_ = − 261, *p* = 0.071). The Darkolivegreen1 (*b*_TE_ = − 369, *p* = 0.001; *b*_IDE_ = − 66, *p* = 0.042; *b*_DE_ = − 303, *p* = 0.008) and Thistle2 (*b*_TE_ = − 373, *p* = 0.002; *b*_IDE_ = − 64, *p* = 0.025; *b*_DE_ = − 309, *p* = 0.010) MEs were also partial statistical mediators of the FEH and right hippocampus volume relationship. The Thistle ME was also a partial mediator in analyses without controlling for TBV (*b*_TE_ = − 382, *p* = 0.007; *b*_IDE_ = − 85, *p* = 0.017; *b*_DE_ = − 297, *p* = 0.042). On the other hand, the Chocolate2, Cornflowerblue, Aliceblue, and Yellow MEs were neither partial nor full mediators of the relationship (p_TE_ < 0.05; p_IDE_ > 0.05; p_DE_ < 0.05). Six of the 11 Burlywood probes are mapped to known genes (*MSH2, ATXN7L1, ODF2, SLC22A6, TGFB3,* and *DYX1C1*) with GO-terms including GO:0005245 voltage-gated calcium channel activity, GO:0002700 regulation of production of molecular mediator of immune response, and GO:0043524 negative regulation of neuron apoptotic process. GO-terms associated with probes from the Darkolivegreen1 and Thistle2 modules include GO:0001829 trophectodermal cell differentiation, GO:0045087 innate immune response, GO:0042552 myelination, and GO:0010506 regulation of autophagy, among others [57, 58].

None of the Darkgray (*b*_TE_ = − 183, *p* = 0.014; *b*_IDE_ = − 47, *p* = 0.095; *b*_DE_ = − 135, *p* = 0.094), Darkolivegreen (*b*_TE_ = − 185, *p* = 0.013; *b*_IDE_ = − 32, *p* = 0.205; *b*_DE_ = − 153, *p* = 0.057), or Lavenderblush2 (*b*_TE_ = − 187, *p* = 0.011; *b*_IDE_ = − 30, *p* = 0.181; *b*_DE_ = − 156, *p* = 0.044) MEs were mediators of the relationship between FEH and left amygdala volume. However, the significant TE values indicated ~ 185 mm^3^ lower left amygdala volume in poor FEH conditions. Regarding FEH and right amygdala volume, Pink4 ME value was a full statistical mediator of the relationship (*b*_TE_ = − 204, *p* = 0.017; *b*_IDE_ = − 48, *p* = 0.026; *b*_DE_ = − 156, *p* = 0.069), indicating that right amygdala volume was 204 mm^3^ less in poor FEH conditions than in high FEH conditions. Results additionally indicate that Pink4 ME value accounted for 48 mm^3^ (24%) of the aforementioned effect. Pink4 was also a full mediator in the non-TBV controlled model (*b*_TE_ = − 208, *p* = 0.017; *b*_IDE_ = − 52, *p* = 0.025; *b*_DE_ = − 157, *p* = 0.087). Pink4 is composed of 21 probes mostly mapped to known genes (*SNORD123, TBCD, FN3K, NRXN3, GLB1L2, SBF2, PSMB1, SYT1, BEST2, TBATA,* and *GNA12*). GO-terms associated with mapped genes include GO:0048487 beta-tubulin binding, GO:0007158 neuron cell–cell adhesion, GO:0007212 dopamine receptor signaling pathway, GO:0010762 regulation of fibroblast migration, GO:0019905 syntaxin binding, and GO:0031683 G-protein beta/gamma-subunit complex binding, among others [57, 58]. Probe-specific genomic biology annotation for partial and full mediating modules can be found in Additional file 1: Table S3. In controlling for 33 tests at FDR = 0.10, all significant ME IDEs, direct effects (DEs), and TEs were BH-significant (Table 3). Mediation analyses were then performed on individual probes from the Pink4 module in order to assess locus-specific effects.

**Table 3:** Module eigengenes (MEs) mediating observed family emotional health (FEH) and brain region volume (BRV) relationships (*n* = 98)

	*b*	95% CI Lower	95% CI Upper
Burlywood: right hippocampus			
Average indirect effect (IDE)	− 111.2*	− 255.5	− 17.9
Average direct effect (DE)	− 254.4*	− 528.2	− 15.8
Average total effect (TE)	− 365.7***	− 641.0	− 151.0
Darkolivegreen1: right hippocampus			
Average indirect effect (IDE)	− 65.6*	− 161.3	− 1.6
Average direct effect (DE)	− 302.9**	− 553.6	− 84.3
Average total effect (TE)	− 368.5**	− 623.6	− 152.7
Thistle2: right hippocampus			
Average indirect effect (IDE)	− 64.4*	− 156.6	− 5.6
Average direct effect (DE)	− 308.5*	− 567.7	− 79.2
Average total effect (TE)	− 372.9**	− 640.1	− 150.5
Chocolate2: right hippocampus			
Average indirect effect (IDE)	− 82.8	− 204.7	4.9
Average direct effect (DE)	− 281.5*	− 585.5	− 24.2
Average total effect (TE)	− 364.3**	− 635.0	− 144.5
Cornflowerblue: right hippocampus			
Average indirect effect (IDE)	− 48.3	− 132.5	14.3
Average direct effect (DE)	− 321.5**	− 606.7	− 96.5
Average total effect (TE)	− 369.8**	− 641.6	− 151.8
Aliceblue: right hippocampus			
Average indirect effect (IDE)	− 53.4	− 146.6	6.6
Average direct effect (DE)	− 317.6**	− 591.1	− 98.8
Average total effect (TE)	− 371.1***	− 642.8	− 162.5
Yellow: right hippocampus			
Average indirect effect (IDE)	− 39.7	− 115.2	14.7
Average direct effect (DE)	− 332.2**	− 610.1	− 113.6
Average total effect (TE)	− 371.9**	− 642.0	− 158.7
Darkgray: left amygdala			
Average indirect effect (IDE)	− 47.4	− 115.8	8.9
Average direct effect (DE)	− 135.2	− 293.1	22.0
Average total effect (TE)	− 182.5*	− 324.2	− 38.6
Darkolivegreen: left amygdala			
Average indirect effect (IDE)	− 32.1	− 89.7	21.8
Average direct effect (DE)	− 152.5	− 310.8	4.6
Average total effect (TE)	− 184.6*	− 323.9	− 39.9
Lavenderblush2: left amygdala			
Average indirect effect (IDE)	− 30.4	− 93.4	13.7
Average direct effect (DE)	− 156.4*	− 301.0	− 3.0
Average total effect (TE)	− 186.9*	− 324.4	− 44.0
Pink4: right amygdala			
Average indirect effect (IDE)	− 47.9*	− 117.4	− 3.7
Average direct effect (DE)	− 156.1	− 338.9	12.9
Average total effect (TE)	− 204.1*	− 380.1	− 44.2

*ME* module eigenvalue, *b* estimate, *CI* confidence interval

***P < 0.001; **P < 0.01; *P < 0.05, P: BH-significant (bolded)

### Probe-wise mediation

Three out of 21 probes from the Pink4 module were full mediators between FEH and right amygdala volume: cg22325292 (*b*_TE_ = − 204, *p* = 0.013; *b*_IDE_ = − 53, *p* = 0.018; *b*_DE_ = − 151, *p* = 0.087), cg02398342 (*b*_TE_ = − 204, *p* = 0.014; *b*_IDE_ = − 44, *p* = 0.038; *b*_DE_ = − 161, *p* = 0.060), and cg00809820 (*b*_TE_ = − 205, *p* = 0.013; *b*_IDE_ = − 48, *p* = 0.049; *b*_DE_ = − 157, *p* = 0.064). These three probes also had extremely high Pearson correlation values with the Pink4 ME (*r* > 0.93, *p* < 2 × 10^–44^), indicating that they are strong representatives of the Pink4 ME. In controlling for 63 tests at FDR = 0.10, all significant probe IDEs, DEs, and TEs were BH-significant (Additional file 1: Table S4).

### Gene set enrichment analysis (GSEA)

We performed GSEA using probe *M*-values as predictors of FEH and used resultant *p* values to facilitate the testing of 3186 GO-terms. After redundancy reduction, 45 BH-significant GO-terms remained for interpretation. CNS-related GO-terms included: beta-amyloid clearance (GO:0097242, *p* = 8 × 10^–11^, rank = 2), filopodia assembly (GO:0046847, *p* = 2 × 10^–8^, rank = 5), catecholamine metabolic process (GO:0006584, *p* = 4 × 10^–5^, rank = 11), and positive regulation of neuron apoptotic process (GO:0043525, *p* = 0.013, rank = 25) among others. Although immune-related terms were limited, one was present in the top three: cytokine receptor activity (GO:0004896, *p* = 8 × 10^–11^, rank = 3). Numerous metabolic functions were identified: negative regulation of stress-activated MAPK cascade (GO:0032873, *p* = 4 × 10^–12^, rank = 1), NAD metabolic process (GO:0019674, *p* = 4 × 10^–9^, rank = 4), and TOR signaling (GO:0031929, *p* = 3 × 10^–4^, rank = 12) among others. A complete list of BH-significant GO-terms can be found in Additional file 1: Table S5.

## Discussion

The current exploratory study examined whether variability in 5mC and fronto-limbic grey matter volume represent pathways through which FEH becomes biologically embedded. Based on previous work, we hypothesized that 5mC modules would be enriched for immune system [14–16], HPA-axis [17, 18], and CNS-relevant [18–22] GO-terms. Our study findings indicated that exposure to poor FEH during childhood was associated with CNS endophenotypes of psychiatric illness, and that a subset of saliva-derived 5mC measurements *statistically* mediated this relationship. Additionally, we found the mediating 5mC modules were enriched with probes associated with GO-terms relating to the CNS and immune system, as well as cellular differentiation, regulation, specialization, and organization (mostly relating to neuronal cell populations). Finally, we found that the underlying FEH-associated methylomic network was enriched with CNS-related, immune system, and metabolic GO-terms. Overall, we posit that the FEH-associated epigenetic signatures could function as proxies of altered fronto-limbic grey matter volume associated with poor childhood FEH; peripheral epigenetic signatures indexing our relationships of interest may be explained by peripheral inflammation related to development of stress-related psychopathology, thereby supporting the neuroimmune network hypothesis [6].

The relationships observed between poor childhood FEH and left/right amygdala volume in the current study mirrored relationships observed throughout the literature regarding direction of effect and magnitude, but not hemisphere-specificity [18, 19]. Studies show hemisphere-specific effects of ASEs on amygdala volume, with stressors exerting notable statistical effects on left but not right amygdala volume. In one such prospective longitudinal study, SLEs negatively predicted left, but not right, amygdala volume in children with low to average polygenic risk scores. They showed that children exposed to the highest level of SLEs had ~ 9% less left amygdala volume than those exposed to the lowest levels of SLEs [18]. A more recent study showed lower left amygdala volumes in children who had experienced early neglect, low SES, or physical abuse compared to non-exposed controls [19]. Although we observed bilateral amygdala grey matter volume associations with poor childhood FEH exposure, our study did show a similar magnitude of effect; poor childhood FEH exposure was explanatory (DE) of − 8.9% difference in left and − 8.4% difference in right amygdala volume. The peripheral 5mC signature (Pink4 ME) mediating right amygdala volume and FEH accounted for − 2.5% of additional volumetric difference (IDE).

Similar to our amygdala-related findings, the reported relationship between poor childhood FEH and low hippocampus volume supports previous findings from the field regarding direction and estimated magnitude of effect, but not hemisphere-specificity [23, 24]. In a prospective longitudinal study, researchers focused on childhood “maternal support” as their exposure of interest, finding that maternal support of children, 3–5 years old, was associated with increased hippocampal volume in both hemispheres later in childhood (7–13 years old). Specifically, they found that children exposed to low maternal support during that time span had a difference in hippocampal volume of − 7.1% [23]. This magnitude closely mirrors the findings of the current study, which show poor childhood FEH has a DE that explains − 6.1% difference in right hippocampal volume, and peripheral 5mC signatures have an IDE responsible for an additional − 1.7% of difference. A more recent study from the same group found that the positive association between SES and hippocampal volume was mediated by “supportive/hostile parenting” in both hemispheres, but only by SLEs in left hippocampus [24]. These studies identified significant associations of maternal support and supportive/hostile parenting in *both* hippocampal hemispheres, whereas the current study identified a significant association only in right hippocampus.

No salient effects of FEH were observed in dlPFC or mPFC, in either hemisphere. This finding does not support research showing deleterious effects of ASEs on frontal cortex morphometry [59–61]. Our findings across fronto-limbic brain regions imply that poor childhood FEH has specific morphometric associations with variability in subcortical structures responsible for memory, avoidance, fear, stress, and negative valence, but not with variability in cortical structures managing those functions.

Beyond the observed associations between poor childhood FEH and fronto-limbic brain morphometry, we were interested in the peripheral epigenetic signatures that index the relationships, and that provide a potential mechanism of biological embedding of ASEs. The Pink4 module, which fully mediated the relationship between poor childhood FEH and right amygdala volume in both TBV-controlled and non TBV-controlled models, is composed of 21 probes, three of which were full mediators of the FEH and right amygdala volume relationship: cg22325292, cg02398342, and cg00809820. Probes cg22325292 and cg02398342 exist in the sixth of six exons of the *FN3K* gene and fall in a putative CpG island and DNaseI hypersensitive region ~ 1000 base pairs upstream of the *TBCD* transcription start site (TSS) [57, 58]. The main *TBCD* protein isomer plays a major role in the assembly of microtubules [62], the cell-cycle progression to mitosis [63], and neuronal morphogenesis [64]. Hypermethylation of the *TBCD* gene in CD4+ T-cells is also associated with rheumatoid arthritis [65], an autoimmune disorder associated with stress exposure [66]. Additionally, cg02398342 falls in the transcription factor binding site of the *EGR1* protein, which has integral, dynamic interactions with genes responsible for vesicular release and endocytosis, neurotransmitter metabolism and receptors, and actin cytoskeleton organization [67]. These interactions facilitate *EGR1*’s significant impact on synaptic and neuronal activation. Module-wide and probe-specific results suggest that the association of poor childhood FEH with right amygdala volume is indexed and *statistically* mediated by peripheral epigenetic signatures relevant to synapse development, neurotransmitter signal transduction, and cytoskeleton organization.

Three modules were partial mediators of the relationship between right hippocampus volume and poor childhood FEH: Burlywood, Darkolivegreen1, and Thistle2. However, the Burlywood module was a full mediator in the non TBV-controlled mediation model, implying that this peripheral epigenetic signature exerts more statistical effect on absolute right hippocampus volume, agnostic of TBV, through poor childhood FEH. GO-terms associated with the hippocampus mediating modules include GO:0002700 regulation of production of molecular mediator of immune response, GO:0043524 negative regulation of neuron apoptotic process, GO:0042552 myelination, and GO:0010506 regulation of autophagy, among others [57, 58]. Module-wide and probe-specific results imply that the associations of poor childhood FEH with right hippocampal volume are indexed by peripheral epigenetics signatures related to immune response and CNS cell development/lifecycle.

The top three GO-terms from our methylome network analysis were: (1) negative regulation of stress-activated MAPK cascade (2) beta-amyloid clearance (3) cytokine receptor activity. The MAPK cascade has long been established as a key driver of eukaryotic signal transduction, but more recently as an integral contributor to cell proliferation, differentiation, and inflammatory processes [68]. There is also a building body of evidence suggesting a significant role of the MAPK cascade in mental health outcomes. In a mouse model, modulation of the MAPK cascade in the forebrain is associated with both anxiety-like and depressive-like behaviors [69]. When p38 MAPK protein is selectively knocked out (KO) of the dorsal raphe nucleus, rodents subjected to social defeat stress show significantly reduced social avoidance compared to wild-type animals [70]. Additionally, pro-inflammatory cytokine administration induces a state of increased serotonergic CNS activity (canonically thought to be depleted in MDD), and induction towards that state is blocked with p38 MAPK inhibition [71]. In humans, MDD is a common co-morbidity of rheumatoid arthritis (RA) [72]; peripheral inflammation is a hallmark of RA and is also observed in MDD patients [73]. Therefore, it is hypothesized that within the context of psychopathology development, environmental stressors induce peripheral cytokine signaling that communicates with fronto-limbic brain regions including the amygdala, hippocampus, and frontal cortex through mechanisms including the MAPK cascade [74]. To this end, numerous RA and anti-depressant drugs are observed to reduce canonical disease symptoms, while also reducing clinical inflammation markers and MAPK signaling [74].

Based on the current study results, it appears that variability in peripheral 5mC and fronto-limbic grey matter volume *potentially* represent pathways through which FEH becomes biologically embedded, with 5mC signatures that mediate the relation between FEH and grey matter volume being especially enriched with GO-terms related to the peripheral inflammatory sequela of stress-related psychopathology development. To our knowledge, the degree to which peripheral 5mC serves as a statistical mediator between poor childhood FEH (or ASEs in general) and variable fronto-limbic brain morphometry had not been previously elucidated. In addition, the observed GO-terms support potential mechanisms of biological embedding that are actively being considered in the field [69–71, 74, 75].

Dimension reduction techniques used throughout our research represent the foremost strengths of this study. These methods focus the analysis onto loci with greater prospect for proxy or surrogate status with etiologically relevant CNS tissue, and reduce the burden of multiple hypothesis testing. Clustering similarly methylated probes creates a relatively small number of modules which potentially contain probes from functionally related genes. On the other hand, limitations of the current study that may reduce the generalizability of the results include retrospective self-report of the main exposure of interest, a relatively small sample size, lack of replication in an independent cohort, the balance of biological sex within the cohort, the cohort enrichment of higher SES and FEH participants, the inability to correct for smoking-related effects, and the interpretation of nominally significant (*p* < 0.05) results instead of more rigorous multiple hypothesis testing corrected results. Additionally, in analyzing grey matter volume of fronto-limbic brain regions as outcomes of interest, we have omitted surface area (SA)- or cortical thickness (CT)-specific effects. The current study also falls short in establishing whether the mediation by peripheral 5mC modules is causal in nature. Longitudinal data could provide more precise insight into whether such relationships exist. Future studies on this topic should capture longitudinal data from a diverse, increased sample size and could investigate genetic factors or tissues of etiological interest.

## Conclusions

The current study showed that, in support of prior literature, exposure to poor childhood FEH is associated with low fronto-limbic BRV as measured in young adulthood. Newly reported here is the finding that saliva-derived 5mC modules mediate the FEH and BRV relationship and are enriched for immune system, CNS-related, and CNS cell development/specialization functions; with additional validation in independent cohorts, these 5mC modules could potentially be used as peripheral biomarkers of poor FEH exposure during childhood. Overall, the findings of the current study support the neuroimmune network hypothesis [6], extend the body of work highlighting neurodevelopmental variability associated with childhood ASE exposure, and inform a potential molecular mechanism of biologic embedding. Future research on these peripheral signatures could validate their use as proxies/biomarkers of perturbed underlying neurobiology in response to poor FEH exposure and could inform further investigation into primarily effected tissue such as endocrine, immune, and CNS cell types.

## Materials and methods

### Participants

The current study draws on data from 98 university students (19.8 ± 1.2 years old; 69% women; 49% white) (mean ± SD) who successfully completed the Duke Neurogenetics Study (DNS). The DNS aims to assess the associations among a wide range of behavioral, neural, and genetic variables in a large sample of young adults, with one of the core goals being to establish a link between these various phenotypes and psychopathology [21, 76–78]. Data from a subset of participants with overlapping demographic, psychosocial, epigenetic, and neuroimaging data were included in the current cohort. This study was approved by the Duke University Medical Center Institutional Review Board, and all experiments were performed in accordance to its guidelines. Prior to the study, all participants provided informed consent. To be eligible for the DNS, all participants were free of: (1) medical diagnoses of cancer, stroke, head injury with loss of consciousness, untreated migraine headaches, diabetes requiring insulin treatment, chronic kidney or liver disease, or lifetime history of psychotic symptoms; (2) use of psychotropic, glucocorticoid, or hypolipidemic medication; and (3) conditions affecting cerebral blood flow and metabolism (e.g., hypertension) [77]. Neither past nor present diagnosis of Diagnostic and Statistical Manual for Mental Disorders, Fourth Edition (DSM-IV) Axis I or select Axis II disorders (borderline and antisocial personality disorders) were exclusion criterion because the DNS seeks to establish broad variability in multiple behavioral phenotypes related to psychopathology [79]. Categorical diagnoses were assessed by trained staff using the electronic Mini International Neuropsychiatric Interview [80] and Structured Clinical Interview for the DSM-IV subtests [81]. Of the total sample reported here, 20 participants (~ 20%) met criteria for at least one lifetime DSM-IV diagnosis (Additional file 1: Table S6).

### Family emotional health (FEH)

Participants were asked to complete the Family History Questionnaire (FHQ), which produced the current study’s measure of FEH. The FHQ is composed *fully* of questions from previously validated inventories [82–88]; of 70 total FHQ questions, 55 were included from the Family History Screen (FHS) [82, 83]. Researchers assessing psychometric properties of the FHS observed specificity in the range of 76.0% (MDD) to 97.1% (suicide attempt), and sensitivity in the range of 31.7% (alcohol dependence) to 60.0% (conduct disorder) [83]. The FHQ and FHS both capture family-wide psychiatric illness, but the FHQ is more encompassing of other ASEs, including 15 questions pertaining to cognitive decline of family members [84], externalizing behaviors [85], exposure to smoking [86], and drug/alcohol abuse treatment [87, 88]. Example questions from the FHQ include, “Has anyone in your family ever felt sad, blue, or depressed for most of the time for 2 weeks or more?”, “Has anyone had several attacks of extreme fear or panic, even though there was nothing to be afraid of?”, and “Has anyone in your immediate family ever tried kill to himself or herself?”. The summed responses from 70 “yes/no” questions based on the aforementioned topics from the FHQ represent the current study’s measure of FEH (Additional file 1: Table S7). Each “no” response corresponded to an additional score of one, with lower values representing poor FEH.

### Cumulative perceived impact of past year stressful life events (past year SLEs)

Participants were administered an inventory measuring the cumulative perceived impact of SLEs from the past year (“past year SLEs”). Prior research reported associations between stress exposure and significant variability in fronto-limbic BRVs [18–20]. Therefore, throughout the current study, we controlled for the effect of past years SLEs using a summation of 45 negatively valenced items [76, 89] from the Life Events Scale for Students (LESS) [90].

### Neuroimaging

ASEs and exposures similar to poor FEH are known to impact fronto-limbic pathways in the human CNS [18–24]. In addition, a meta-analysis has shown that both the hippocampus and amygdala have hemisphere-specific volume differences in healthy adults [91], and ASEs are known to have hemisphere-specific effects on fronto-limbic brain regions [24, 92]. Due to the dearth of research surrounding the potential effects of 5mC and ASEs (independently or in causal pathway models) on fronto-limbic brain region volume variability, and in order to avoid omission of hemisphere-specific effects by taking the mean of hemisphere volumes, hemisphere-specific amygdala, hippocampus, dlPFC, and mPFC volume measures were analyzed. Volume measurements of dlPFC and mPFC were chosen as outcome variables from the frontal cortex due to the opposing nature of their afferent and efferent projections to hippocampus and amygdala, and their functional relationships with each region [8]. To compare differences between hemisphere volumes of mPFC, dlPFC, amygdala, and hippocampus, we performed paired sample *t*-tests of each brain region.

Data were collected at the Duke-UNC Brain Imaging and Analysis Center using one of two identical research-dedicated GE MR750 3T scanners (General Electric Healthcare, Little Chalfont, United Kingdom) equipped with high-power high-duty cycle 50-mT/m gradients at 200 T/m/s slew rate, and an eight-channel head coil for parallel imaging at high bandwidth up to 1 MHz. T1-weighted images were obtained using a 3D Ax FSPGR BRAVO with the following parameters: TR = 8.148 ms; TE = 3.22 ms; 162 axial slices; flip angle, 12°; FOV, 240 mm; matrix = 256 × 256; slice thickness = 1 mm with no gap; and total scan time = 4 min and 13 s. To generate regional measures of brain morphometry, anatomical images for each subject were first skull-stripped [93], then submitted to Freesurfer's (Version 5.3) “recon-all” with the “noskullstrip” option [94, 95], using an x86_64 linux cluster. CT and SA for 31 regions in each hemisphere, as defined by the Desikan-Killiany-Tourville atlas [96], were extracted using Freesurfer. Additionally, gray matter volumes from eight subcortical regions (including hippocampus and amygdala) were extracted with Freesurfer's subcortical segmentation (“aseg”) pipeline [97], along with estimated TBV. The gray and white matter boundaries determined by recon-all were visually inspected using FreeSurfer QA Tools and determined to be sufficiently accurate for all subjects.

### Molecular

Saliva was collected from participants using the Oragene-DNA OG-500 kit (Oragene; Ottawa, Canada). DNA was extracted and cleaned using the DNA Genotek prepIT PT-L2P kit (DNA Genotek Inc; Ottawa, Canada) using manufacturer recommended methods. Purity of extracted DNA samples was assessed by absorbance using Nanodrop 1000 spectrophotometer (Thermo Fisher Scientific Inc; Waltham, Massachusetts). The quantity of double-stranded DNA was assessed using Quant-iT PicoGreen dsDNA kits with manufacturer recommended protocols (Invitrogen; Carlsbad, California). A total of 500 ng of genomic DNA was bisulfite-converted (BSC) using manufacturer-recommended EZ DNAm kits (Zymo Research; Irvine, California). After conversion, BSC DNA was applied to the Infinium MethylationEPIC BeadChip (Illumina; San Diego, California) (850 k) to measure 5mC at ~ 850 k loci.

### 5mC pre-processing

Beta-values measured from the 850 k platform were background corrected in GenomeStudio, quality controlled, and filtered according to previously published methods [98]. All quality control and pre-processing was performed in R, version 3.6.1 [99]. These steps removed 112,307 low quality and potentially cross-hybridizing probes, quantile-normalized probe beta-values, and removed potentially confounding technical and batch effects. 5mC beta-values were variance stabilized and logit-transformed into M-values [100]. 15,063 X-chromosome, Y-chromosome, and *rs*-mapped probes were removed to focus the analysis on genomic loci common between both biological sexes. The remaining ~ 739 k probes were then subset to include only those with observed significant Pearson correlation (*p* < 0.05) between saliva and brain tissue from the ImageCpG data repository [101]. This was done to focus the analysis on loci with greater prospect for proxy or surrogate status with etiologically relevant CNS tissue. Probes removed during pre-processing and subsetting were not analyzed. Afterwards, 62,422 probes remained for following analyses.

### Cellular heterogeneity

Cell heterogeneity was estimated using a reference-free deconvolution method [102, 103]. Briefly, the top 15 k most variable CpG sites were selected from the pre-processed/quality controlled 850 k data and used to estimate the number of cell types and generate a matrix containing the proportions. Based on these methods, the number of cell types was set at five. Estimated proportions were used as covariates in relevant analyses to account for cellular heterogeneity.

### Genomic ancestry

To avoid potential inaccuracies and confounding effects of self-reported race/ethnicity, genetic ancestry was modeled using multi-dimensional scaling (MDS) measures extracted from participant genomic data using PLINK [104]. Using previously collected GWAS data from the DNS, the first four MDS genetic ancestry measures were calculated and used as covariates across pertinent models based on visual inspection of scree plots. This methodology is in line with previous publications [77].

### Probe clustering

To remove non-desired effects, we fit linear models with age, validated biological sex, cellular heterogeneity, and genomic ancestry as predictors of probe-wise 5mC *M*-value. For each probe, residual values (“residualized *M*-values”) were extracted for clustering. Taking the 62,422 residualized *M*-values, the “WGCNA” R package was used to build a co-methylation network [105]. First, scale-free topology model fit was analyzed. As recommended, a soft-threshold value of four was chosen based on the lowest power at which adjusted *R*^2^ > 0.90. Adjacency and dissimilarity matrices were generated, and unsupervised hierarchical clustering was used to generate a clustered, residual M-value network. Setting a minimum cluster size of 10 generated 194 modules, each identified by a unique color. The ME of each module was then calculated. Compared to epigenome-wide association studies, which assess differential methylation on the level of individual 5mC loci, network-based methods, as used in the current research, utilize dimension-reduction techniques to create a much smaller network of related 5mC probe clusters. This reduces the burden of multiple hypothesis testing, and provides the potential for increased statistical power in circumstances with a small number of biological replicates [106].

### Statistical analyses

**Fig. 3:**
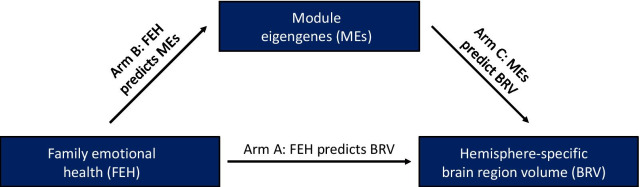
Conceptual model testing module eigengenes (MEs) as mediators of the hypothesized association between family emotional health (FEH) and variability in hemisphere-specific brain region volume (BRV). **Arm A.** FEH was used as a predictor of hemisphere-specific BRV, while including age, biological sex, four genomic ancestry MDS measures, past year SLEs, and total brain volume as covariates. **Arm B.** FEH was used as a predictor of ME value, while including past year SLEs as a covariate. The age, sex, and genomic ancestry effects on ME components were previously removed. **Arm C.** ME values were used as individual predictors of BRV, while including age, biological sex, four genomic ancestry MDS measures, past year SLEs, and total brain volume as covariates

In order to understand the relationships between variables, we computed Pearson correlations and mapped their correlation coefficients. Based on these correlations, we conducted a set of analyses, as shown in Fig. 3. In Arm A analyses, FEH was used as a predictor of hemisphere-specific BRVs, while including age, biological sex, four genomic ancestry MDS measures, past year SLEs, and TBV as covariates. In Arm B analyses, FEH was used as a predictor of ME values, while including past year SLEs as a covariate. Age, sex, and genomic ancestry effects were accounted for previously by using residualized M-values as input for clustering. In Arm C analyses, ME values were used as individual predictors of BRV, while including the same covariates as in Arm A. Throughout the current research, past year SLEs were included as a covariate because our FEH measure only captures SLEs from childhood, and recent stress exposure is associated with variability in our outcome variables [18, 19, 24, 107]. TBV was included as a covariate but, where pertinent, non-TBV controlled model results are also reported. Within each phase of the analyses, non-standardized continuous measures were used resulting in non-standardized effect estimates. Due to the exploratory nature of the current study, dependent variables were graduated to subsequent study arms if *p* < 0.05; sequential BH-significant results at FDR = 0.10 are also reported where applicable [108], and all results with *p* < 0.05 were considered for interpretation. Briefly, for each *p* value, a BH critical value was calculated where the *p* value’s assigned rank over the number of tests was multiplied by the accepted FDR. *p* values less than this threshold were deemed BH-significant.

### Mediation analyses

To investigate whether the effect of poor FEH on hemisphere-specific BRV is *statistically* mediated via peripheral 5mC signatures, MEs were tested for mediating status between FEH and hemisphere-specific BRVs using the “mediation” package in R [109] (Fig. 3). Importantly, only hemisphere-specific BRVs associated with FEH (Fig. 3, Arm A) were considered. Similarly, MEs tested for mediation included *only those* associated with both FEH (Fig. 3, Arm B) and hemisphere-specific BRV (Fig. 3, Arm C). Mediation model inputs were assembled per recommended “mediation” package protocol. Therefore, Arm A (plus ME as a covariate) and Arm B models were used as inputs. For each ME, indirect effects (IDE), direct effects (DE), and total effects (TE) were calculated as a result of 10,000 non-parametric bootstrap simulations. Consistent with published methods [24], we considered an ME a full mediator if the DE = 0 while the IDE and TE ≠ 0, or a partial mediator if the DE, IDE, and TE ≠ 0. Individual probes from full mediator modules were assessed for mediation status as well.

### Gene set enrichment

To assess the underlying methylomic network enrichment of the ~ 62,000 brain-saliva correlated probes, individual residualized probe M-values were used as predictors of FEH in Bayesian regression models. Age, sex, genomic ancestry measures, cell heterogeneity measures, and past year SLEs were included as covariates. From this analysis, BH-significant probe *p* values were extracted and used as input to GSEA in the “methylGSA” package [110]. GO sets composed of 50 to 1000 genes were allowed, which eliminated high-level GO-terms such as “biological process” and facilitated testing of 3186 GO sets. To produce a condensed summary of non-redundant GO-terms, the web-based tool “Revigo” was used [111].

## Supplementary Information

**Additional file 1: Supplementary Tables**. **Table S1**. FEH predicts MEs. **Table S2**. MEs predict BRV. **Table S3**. Probe specific annotations. **Table S4**. Probe-wise mediation. **Table S5**. Condensed GSEA. **Table S6**. Cohort-level mental health diagnoses. **Table S7**. FHQ questionnaire

## Data Availability

The ImageCpG dataset supporting the conclusions of this article is available at Gene Expression Omnibus (GEO) Accession GSE111165; http://han-lab.org/methylation/default/imageCpG#. Due to the provisions of the informed consent documents for this study, individual-level DNS data cannot be posted publicly; however, these data may be obtained by investigators approved through the following procedures: http://www.haririlab.com/projects/procedures.html.
